# Relation between *Plasmodium falciparum *asymptomatic infection and malaria attacks in a cohort of Senegalese children

**DOI:** 10.1186/1475-2875-7-193

**Published:** 2008-09-29

**Authors:** Agnès Le Port, Michel Cot, Jean-François Etard, Oumar Gaye, Florence Migot-Nabias, André Garcia

**Affiliations:** 1 Institut de Recherche pour le Développement (IRD), Unité de Recherche 010: Santé de la mère et de l'enfant en milieu tropical. Laboratoire de Parasitologie, Faculté de Pharmacie, 4 avenue de l'Observatoire, 75006 Paris, France; 2 IRD, UMR 145 ≪ VIH/sida et maladies associées ≫, HCL – Service de Biostatistique, 162 Avenue Lacassagne, 69424 Lyon, Cedex 03, France; 3 Laboratoire de Parasitologie et de Mycologie, Département de Biologie et d'Explorations fonctionnelles, Faculté de Médecine, Université Cheikh Anta Diop (UCAD), Dakar, Sénégal; 4 IRD, UR010: Santé de la mère et de l'enfant en milieu tropical, 08 BO 841, Cotonou, Benin

## Abstract

**Background:**

It is important to establish whether or not the presence of malaria parasites in peripheral blood of asymptomatic individuals is a predictor of future clinical mild malaria attacks (MMA). The aim of this study was to determine how an asymptomatic positive thick blood smear could be related to the occurrence of a MMA during the nine following days.

**Methods:**

The study was conducted in a cohort of 569 Senegalese children, who were investigated for *Plasmodium falciparum *asymptomatic carriage at two different times of the transmission season, the beginning (September) and the end (November). The occurrence of MMA was investigated in asymptomatic carriers and non-carriers, every three days for nine consecutive days. Survival analysis was performed and risk estimates were calculated by Cox proportional hazards model.

**Results:**

At the beginning of the transmission season, 27.8% (147/529) of the children were asymptomatic carriers (ACs) and 5.4% (8/147) of MMA occurred among these, versus 1% (4/382) among non-carriers (RR = 5.32; IC = [1.56–18.15], p = 0.008). At the end of the transmission season, the frequency of asymptomatic carriers was similar to that observed at the beginning of the season (31.9%, p = 0.15), but no MMA was detected during this period.

**Conclusion:**

A significant association between *P. falciparum *asymptomatic carriage and the occurrence of MMA at the beginning of the transmission season was demonstrated, with a five-fold increase in the risk of developing a MMA in ACs. In the context of a possible distribution of IPTc in the future, drug strategies may have dramatic consequences due to the existence of ACs (both long term and short term), as they seem to play an important role in the individual protection to malaria, in the most exposed age groups.

## Background

*Plasmodium falciparum *asymptomatic carriers (ACs), i.e., individuals harbouring parasites without clinical signs, are numerous in areas of high transmission. The consequences and significance of such asymptomatic infections have both been studied in diverse situations and from complementary approaches, but these studies led to contradictory results [1-4]. According to a few authors, long term asymptomatic carriage may represent a form of tolerance to the parasite in children building up their immune response. In this way, asymptomatic carriage would protect these children from developing either a mild malaria attack (MMA) or a more severe one, by keeping their immunity effective [1-3]. Conversely, asymptomatic carriage may represent a mode of entry to symptomatic malaria, especially in young children [4,5].

It is important to understand the process which leads some of these children to suddenly develop a MMA. The time course of the relation between *Plasmodium falciparum *infection and MMA occurrence needed to be investigated [5]. If the clinical outcome of infection can be determined by the host's ability to regulate the parasite growth over time, the way by which this regulation prevents the disease is incompletely known [3,6,7]. Investigating this issue other important factors have to be considered, such as the age of exposed children, or the multiplicity of infections by different plasmodial populations in a single individual [1-4].

Treatment of asymptomatic individuals, regardless of their malaria infection status, with regularly spaced therapeutic doses of antimalarial drugs has been proposed as a method to reduce malaria morbidity and mortality [8]. This strategy, called intermittent preventive treatment (IPT), is currently employed for pregnant women (IPTp) and is being studied for infants (IPTi) and children (IPTc). The effects of repeated treatments on the development of immunity are the major challenges of intermittent preventive treatment [9] and it is of great importance to increase the knowledge on the asymptomatic carriage of malaria parasites in order to help to assess the risk/benefit ratio of such new strategies.

To evaluate how asymptomatic carriage could be related to the occurrence of uncomplicated malaria attacks, a follow-up of a cohort of Senegalese children was carried on in an area of marked seasonal transmission. To determine the variations of the balance between clinical signs and the absence of symptoms during the transmission season, a survey on the same population at the beginning and at the end of the transmission season was performed (i.e. September and November 2002).

## Methods

### Study sites

This study was carried out during the 2002 malaria transmission season, in two villages of the Niakhar area (Diohine and Toucar), located 150 km south-east of Dakar [10]. The climate is characteristic of a sahelian region with distinct rainy (from July to September) and dry seasons (October to June). Malaria transmission is seasonal, from the middle of August until the end of November, with a peak in September. Transmission has been estimated between nine and 12 infective bites per person and per year, exclusively by mosquitoes of the *Anopheles gambiae s.l. *complex, mainly by *Anopheles arabiensis *[10,11]. Pluviometric data collected by an IRD team in 2002 showed that the two villages were equivalent in terms of numbers of rainy days (5/14 days) and of quantity of water fallen (6.12 mm at Diohine and 5.19 mm in Toucar, p = 0.80) during the two weeks preceding the study.

### Population study and study design

566 children and adolescents, from two to 17 years of age, were investigated twice during the study: at the beginning (September 2002) and at the end (November 2002) of the transmission season. All eligible children in the area were asked, at each period, to attend the health centre where a systematic thick blood smear was performed. A urine sample was collected in order to check for the presence of chloroquine metabolites. Children were classified according to their clinical and parasitological status: (1) children harbouring a *P. falciparum *parasitaemia without fever or clinical signs were classified as asymptomatic carriers (ACs); (2) children with a negative thick blood smear were classified as non-carriers, and (3) children presenting with a clinical malaria attack (see below) were treated and further excluded from the analysis.

An active survey of clinical malaria attacks was set up. Children were visited at home every three days by investigators recording axillary temperature. In case of temperature exceeding 37.5°C or fever reported by the parents since the last visit (or other symptoms such as headache, vomiting and weakness), a thick blood smear was performed. Parents were urged to bring their child to the health centre between the scheduled visits if any kind of symptom appeared. A MMA was defined as the association of a temperature over 37.5°C (or reported fever between two visits) and a parasite density over 2,500 trophozoites/μl of blood. The same threshold had been previously used in various studies performed among populations living in the Niakhar area [12-14]. At each transmission period, we performed this three-day periodic control of clinical malaria attacks for a whole duration of the nine days following the date of the systematic thick blood smear. A nine days follow-up was undertaken, which is the time period corresponding to approximately four life cycles of *P. falciparum*, in order to minimize the probability to deal with reinfections [15].

When a clinical malaria attack was suspected, a presumptive treatment was administered according to the "Programme National de Lutte contre le Paludisme" (PNLP) recommendations. This protocol was approved by the ethics committee of the Health Ministry of the Republic of Senegal (N°000526/MS/DERF/DER).

### Laboratory methods

*Plasmodium falciparum *asexual blood forms were counted on Giemsa-stained smears, using a previously described method [13]. A blood smear was declared negative when no parasite was detected in 200 fields. The presence of chloroquine in urine samples was assessed by a colorimetric method (Haskins modified by Mount, HMM II), and positive results (≥ 3 μg/ml) gave information about previous intake of antimalarial drugs [16].

### Measured covariates

For each child the following covariates were collected: (1) sex; (2) age; (3) village of residence and (4) presence of chloroquine metabolites in urine (≥ 3 μg/ml).

### Statistical procedure

The same statistical strategy was applied during the two periods. We first performed an univariate analysis to test for associations between the occurrence of MMA (yes/no), asymptomatic carriage (yes/no), and measured covariates. Pearson's chi-square and one-way analysis of variance were used for qualitative and quantitative variables respectively. When non parametric methods were required, Fisher's exact test or Kruskall-Wallis test were applied. Factors associated with asymptomatic carriage and MMA occurrence were selected at a p value < 0.15.

The delay between the systematic parasitological measurement and the appearance (or not) of a MMA during the nine following days was analysed, using survival time data procedures.

- The Kaplan-Meier survival analysis and the Log-Rank test were used to compare incidence rates of MMA between ACs and non-carriers.

- The Cox proportional hazards model was used to analyse the time to development of a MMA in each group, adjusted on the covariates found significant (p < 0.15) in the univariate analysis.

**Figure 1:**
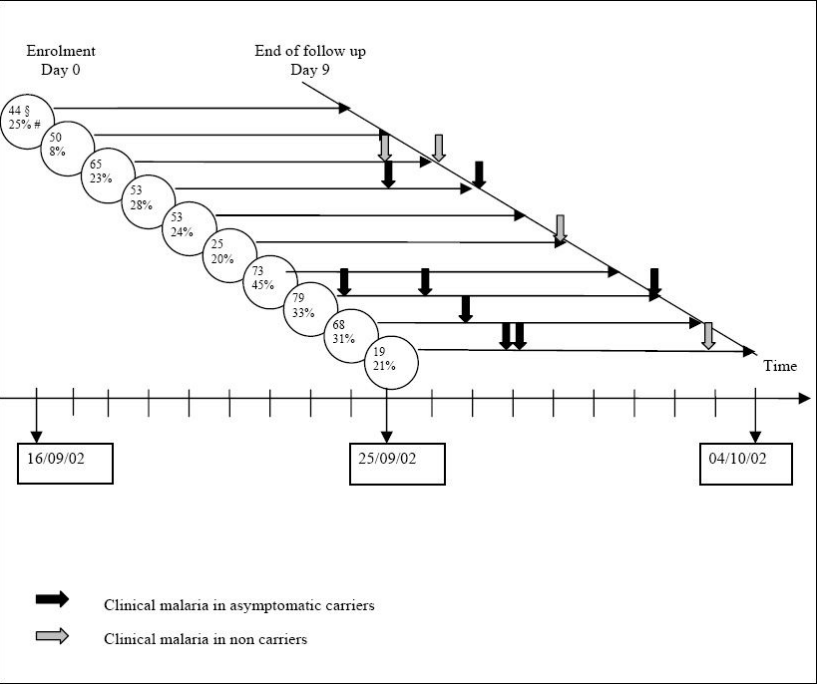
**Clinical malaria occurrences over time at the beginning of the malaria transmission season, Niakhar, 2002**. § n: number of patients per day. # PI: Plasmodial Index (= ACs frequency).

Survival times were estimated as follows (Figure 1): the baseline (time 0) was defined as the date of enrolment (date of systematic thick blood smear); the time to first MMA was defined as the interval between the baseline date and the MMA occurrence date. Subjects who presented no MMA during the follow-up were censored at the endpoint, at Day 9. Proportional Hazard (PH) and log-linearity hypotheses were tested. For all analyses, a p value below 0.05 was considered significant. The statistical analyses were performed using Stata v. 6.0.

## Results

### Study population

A total of 529 and 527 children from the original 566 were enrolled respectively at the beginning and at the end of the transmission season. The mean age (SD) of the population was 8.6 (0.15) years and the sex-ratio (male/female) was 1.35. Fifty seven per cent of children belonged to the village of Diohine and forty three percent to the village of Toucar. Children who did not attend the health centre in September (n = 37) and in November (n = 39) were significantly older than children who did. Activities such as farm work or looking after livestock may explain this difference.

### Beginning of the transmission season

In September, 27.8% (147/529) of the children were asymptomatic carriers (Figure 1). The mean parasite density was 7,169.4 parasites/μl of blood (SD = 34943.8). No differences on mean parasite density according to the day of enrolment were observed (p = 0.73). Plasmodial index varied from one day to another (p < 10^-3^), bur without any particular trend of increase. The proportion of AC did not differ (p > 0.70) between children with CQ metabolites in their urine (9/36; 25%) and others (129/468; 27.6%). No child was lost to follow-up. During the nine following days, 2.3% (12/529) of the whole group presented a MMA, i.e. 5.4% (8/147) of the ACs and 1.0% (4/382) of the non carriers. The mean parasite density was 34,243.3 parasites/μl of blood (SD = 31,460.6), extremes values going from 4,040 to 108,800 parasites/μl of blood. The information concerning the use of impregnated bednets was collected. It appears that 7.52% of asymptomatic carriers reported the use of these bednets, vs 5.38% among non carriers, p = 0.624. All children who presented a mild malaria attack were not sleeping under impregnated bednets (unknown data for 2 children in this group). Information concerning the treatment delivered in the area by the dispensaries during the study was collected by our team and we didn't notice differences among villages.

### Association between asymptomatic carriage and MMA occurrence

Associations between MMA, asymptomatic carriage, and measured covariates (age, sex, village and chloroquine in urine) in univariate analysis are shown in Table 1. There was a significant association (p = 0.005) between *P. falciparum *asymptomatic carriage at inclusion and the occurrence of a MMA during the nine following days. Village of residence (p < 0.0001) and, in a lesser extent, sex (p = 0.06) were associated with asymptomatic carriage. Age was neither associated with MMA nor asymptomatic carriage. However because of its importance, it was included in multivariate analysis as well.

**Table 1:** Association between *P. falciparum *asymptomatic carriage or clinical malaria occurrence and other covariates at the beginning of the malaria transmission season, Niakhar, 2002.

	MMA			*P. falciparum *asymptomatic carriage			
	Number	(%)	p value*	Number	(%)	P value	Total Number
*P. falciparum *asymptomatic carriage							
Yes	8	5,4	0,005				147
No	4	1,0					382
Age (years)							
[2–5]	3	2,6	0,93	29	25,4	0,56	114
[6–10]	5	2,1		64	26,8		239
[11–17]	4	2,3		54	30,7		176
Sex							
M	7	2,3	0,95	94	30,9	0,06	304
F	5	2,2		53	23,6		225
Village							
Diohine	5	1,7	0,34	63	21,5	< 0,001	292
Toucar	7	2,9		84	35,4		237
Chloroquine in urine ^a^							
Yes	0	0	-	9	25	0,74	36
No	12	2,6		129	27,6		468

^a ^Data available for 504 children

### Survival analysis

**Figure 2:**
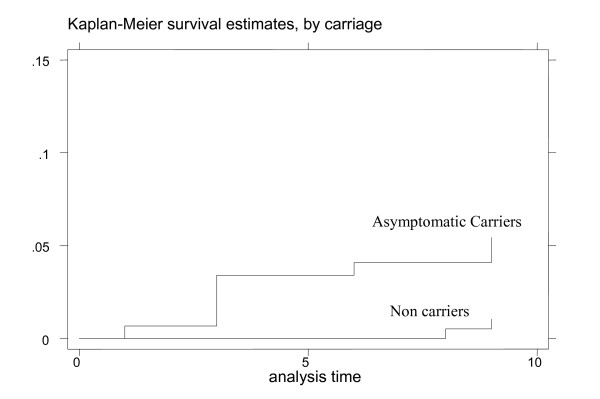
MMA rate estimates in ACs and non carriers by the Kaplan Meier method at the beginning of the malaria transmission season, Niakhar, 2002.

The rate of MMA occurrence was significantly higher over the nine days of follow-up in ACs compared to non carriers (6.8% vs. 1.6%, p = 0.002) (Figure 2).

### Multivariate analysis (Cox model)

*Plasmodium falciparum *asymptomatic carriage was predictive of a MMA occurrence during the nine following days. *Plasmodium falciparum *ACs had a five-fold increased risk of MMA compared to non carriers (RR = 5.32, IC = [1.56–18.16], p = 0.008) (Table 2). Age, sex and village did not remain significant after adjustment. The proportional hazard assumption was respected according to global and graphical tests.

**Table 2:** Cox multivariate analysis: time to first MMA in relation with *P. falciparum *asymptomatic carriage and other covariates at the beginning of the malaria transmission season, Niakhar, 2002.

	RR (95% CI)	P value
Asymptomatic carriage		
No^a^	1	
Yes	5.32 [1.56–18.16]	0.008
Age		
[2–5]^a^	1	
[6–10]	0.76 [0.18–3.18]	0.704
[11–17]	0.82 [0.18–3.71]	0.798
Gender		
F^a^	1	
M	0.84 [0.26–2.74]	0.778
Village		
Diohine^a^	1	
Toucar	0.58 [0.04–8.28]	0.690

^a ^Reference class

### End of the transmission season

**Figure 3:**
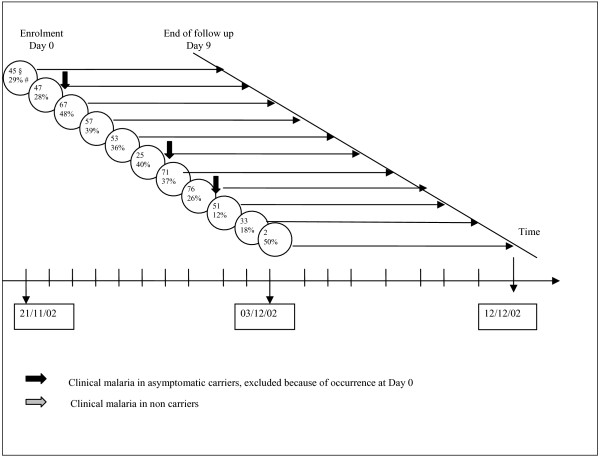
**Clinical malaria occurrences over time at the end of the malaria transmission season, Niakhar, 2002**. § n: number of patients per day. # PI: Plasmodial Index (= ACs frequency).

527 children out of 566 were present at the end of the malaria transmission season (Figure 3). Three children presented a MMA on the day of enrolment and were excluded. The remaining 524 children did not differ from those included in the first study period for age, sex ratio, village of residence and frequency of positiveness for the search of chloroquine metabolites in the urine. The mean parasite density at enrolment was 5,597.6 parasites/μl (SD = 13,941.1) and was not different from the beginning of the transmission season (p = 0.14). The frequency of *Plasmodium falciparum *ACs was similar at enrolment between the two periods (27.8% at the beginning vs. 31.9% at the end of the transmission season, p = 0.15). The parasite density was homogenous during the 10 days of enrolment (p = 0.13), but not the plasmodial index (p = 0.005), even if there was no particular trend of increase. The same pattern of results concerning the association of AC and measured covariates as observed in September, was observed at this period. No MMA occurred during the nine following days.

## Discussion

This study demonstrated that, in a cohort of Senegalese children, there was a significant association between *P. falciparum *asymptomatic carriage and the occurrence of MMA. The relation was true at the beginning (September), but not at the end of the transmission season (November). In the first period, children harbouring *P. falciparum *had a five-fold increased risk to develop a MMA during the nine following days, independently of their age.

*Plasmodium falciparum *asymptomatic carriage concerns a very important proportion of exposed populations in endemic areas. However, the accurate definition of asymptomatic carriage relative to its duration (i.e. long term or short term) differs from one study to another. In the present work it was intended to minimize the probability to deal with reinfections and a short term follow-up of nine days was chosen, as done by Bouvier *et al *in a previous study [15]. The definition of a case of malaria is also important. Bouvier, whose protocol was a close follow-up including daily fever survey, showed in Mali an association between AC and the occurrence of fever during the nine following days, which depended on both season and age [15].

To see if there was a similarity with the results of Bouvier, the survival analysis was performed using different diagnosis criteria for MMA (temperature over 37.5°C associated with a positive blood smear regardless of parasite density). The main results of the study were confirmed with these different criteria, both at the beginning and at the end of the transmission season.

The results of the present study show the absence of effect of age on children susceptibility to present a MMA, which is an important individual parameter concerning the acquisition of immunity. They differ from a recent study performed in Tanzania, which evidenced an age-dependent risk of clinical malaria associated with asymptomatic carriage [4]. In this latter study, at the beginning of the follow-up, parasitaemic children below one year of age had a three-fold increase in clinical malaria incidence, compared to aparasitaemic children of the same age. In older children, baseline parasitaemia appeared to be a protective factor. The authors suggested that repeated *P. falciparum *infections induced a protective response in older children regularly exposed with less subsequent morbidity. Conversely, in young children with low previous exposure, recent malaria infection represented a higher risk of clinical attacks [17,18].

Differences in the levels of malaria transmission between the two study areas (Tanzania and Senegal) can be an explanation to the observed discrepancies. Transmission is intense and perennial in Tanzania, while in Niakhar it is low and strongly seasonal. Moreover, in the study period (2002), the overall rainfall level in Niakhar was notably lower than the mean rainfall over the past 16 years (1984–2000) [11]. It can thus be considered that children from Niakhar were less exposed to infection and hence, their susceptibility to malaria was maintained, contrary to Tanzanian children who probably had acquired an earlier protective immunity.

Differences observed between the beginning and the end of transmission season could be explained by the hypothesis that immunity to uncomplicated malaria symptoms, which requires an important number of infections over many years to become established, can be partitioned in two components: an immunity refraining the outbreak of symptoms (clinical immunity) and an immunity allowing to control parasite density (infection immunity) (reviewed in [19]). The ability to control symptoms develops before the ability to control parasite replication, as suggested by the rate of parasite density required for the symptoms to appear [20-22]. However, immune mechanisms allowing the control of parasite growth may remain active in the absence of re-infections during the dry season (from January to September), contrary to clinical immunity, which could be quickly lost in the absence of stimulation [19]. The results of the present study could be explained by a rapid loss of the immune mechanisms involved in clinical immunity during the nine month-long dry season in the absence of re-infection. Consequently, children infected early at the beginning of the transmission season in September presented an increased risk to develop rapidly a MMA. Then, after repeated infections, the majority of them possibly recovered an efficacious clinical immunity, explaining the absence of MMA in November.

Moreover, the multiplicity of infections (MOI) by different plasmodial strains can bring a complementary explanation to the differences found in the study between the beginning and the end of the transmission season. The role of MOI on malaria morbidity has recently drawn a lot of attention but several contradictory results have been published. Mayor *et al *have shown that multiple infections are associated with an increased number of clinical episodes in very low transmission areas [23]. Conversely, several studies on partially immune children have found opposite results in areas of high [1,3] as well as low-to-moderate but perennial transmission [2]. In the Senegalese area, the characterization of parasites, based on *msp2 *genotyping, was performed in June 2002 (before the transmission season) and January 2003 (at the end of the transmission season) in a cohort of 400 children living in the same villages as the children enrolled in the present study. A significant increase of the mean value of MOI was found between June 2002 and January 2003, i.e. 2.5 and 4.1 respectively (p < 10^-4^) [24]. These results are consistent with the idea that the emergence of new *Plasmodium falciparum *genotypes during the transmission peak in September can be associated with an increased incidence of MMA. Later in the transmission season, children may have recovered their clinical immunity quickly as they have been accumulating infections by various parasite strains.

Treatment of asymptomatic individuals with regularly spaced therapeutic doses of antimalarial drugs, called IPT, has been proposed as a method to reduce malaria morbidity and mortality. At the same period and in the same area, a clinical trial was undertaken by Cisse *et al *to determine the efficacy of IPTc to protect children from presenting a MMA. IPTc was distributed to children from two to 59 months living in villages surrounding the two villages of the present study, at three periods of the transmission season. Doses where delivered in September (beginning of the transmission season), October (four weeks after the first dose) and November (end of the transmission season) and they obtained high degrees of protection against clinical malaria, with a 90% decrease in MMA incidence [25]. Such high levels of protection were not confirmed by other studies realised in Mali [26] or Ghana [27].

The results of the present study lead to interrogations. In the case of a systematic distribution of IPTc by health workers at the beginning of the transmission season, all children would be treated independently of their parasite carriage, i.e. children at risk at the beginning and protected at the end of the transmission season (ACs) as well as non carriers, protected at the end of the season. Immunological protection against malaria would be somehow replaced by chemical protection. The consequence of an interruption of IPT distribution, due to low availability of the drugs or dysfunction of the distribution process would be an incomplete coverage of chemical protection during the transmission season. Supposing the second dose, given in October, was not distributed, the situation would be similar to the beginning of the transmission season as described in the present study. Children could present a MMA at the end of the transmission season, with no noticeable benefit due to IPT.

A recently published study on the same population has focused on long term asymptomatic carriage [28]. ACs were detected in June 2002 and it was demonstrated that asymptomatic carriage during the dry season was a protective factor against MMA during the next transmission season. These results were confirmed in the following year and among the long term ACs defined in 2002, 90 children were also found to be ACs in 2003. These very particular children were denoted as potentially protected. ACs who presented a MMA in the present study were not belonging to this group of 90 long term ACs, suggesting the existence of two different types of ACs: short term exposed to clinical malaria and long term presumably protected.

The long term *P. falciparum *carriage would be suppressed, as well as short term carriage, in the event of IPT distribution. Thus, the consequences of a shortage of drugs would be dramatic, as potentially increasing the population of susceptible children. The existence of ACs, both short term and long term, stresses the atmost importance of a perfect coverage of the transmission season by IPT, with sufficient drug supplies and well motivated health workers, as explained by Greenwood [29].

## Conclusion

In a seasonal marked malaria transmission area, asymptomatic carriers are at risk of presenting a MMA at the beginning of the transmission season, and not at the end. Such differences observed between the two periods within the transmission season can be related both to the acquisition of immunity and the evolution of the genetic diversity of parasite strains in the course of the transmission season. In the future, it may be important to assess epidemiological or interventional studies in transmission area, taking into account the period within the transmission season. The interest on asymptomatic carriage should not be abandoned, as it may be a form of protection at some moments of the year. The probable existence of several types of asymptomatic carriage (i.e. short term and long term) and their characteristics have to continue to be investigated, at the time when intermittent preventive treatment for infants (IPTi) and children (IPTc) is under evaluation and could be applied generally.

## List of abbreviations

AC: asymptomatic carrier; MMA: mild malaria attack; MOI: multiplicity of infection.

## Competing interests

The authors declare that they have no competing interests.

## Authors' contributions

ALP participated in the collection of data, performed the statistical analysis and drafted the manuscript. MC participated to in the design of the study, helped to draft the manuscript and revised the paper critically. FMN participated in the design and coordination of the study, the collection of data and made helpful comments on the manuscript. JFE and OG participated in design of the study and revised the paper. AG was the conceptor of the study, participated to its design and coordination, organized the collection of data, helped to draft the manuscript and revised it. All authors read and approved the final manuscript.
